# The JAK1 Selective Inhibitor ABT 317 Blocks Signaling Through Interferon-γ and Common γ Chain Cytokine Receptors to Reverse Autoimmune Diabetes in NOD Mice

**DOI:** 10.3389/fimmu.2020.588543

**Published:** 2020-12-04

**Authors:** Tingting Ge, Gaurang Jhala, Stacey Fynch, Satoru Akazawa, Sara Litwak, Evan G. Pappas, Tara Catterall, Ishan Vakil, Andrew J. Long, Lisa M. Olson, Balasubramanian Krishnamurthy, Thomas W. Kay, Helen E. Thomas

**Affiliations:** ^1^ Immunology and Diabetes Unit, St Vincent’s Institute, Fitzroy, VIC, Australia; ^2^ Department of Medicine, St Vincent’s Hospital, The University of Melbourne, Fitzroy, VIC, Australia; ^3^ AbbVie Bioresearch Center, Worcester, MA, United States

**Keywords:** JAK-STAT signaling pathway, cytokines, non-obese diabetic mouse, CD8+ T cell, type 1 diabetes mellitus

## Abstract

Cytokines that signal through the JAK-STAT pathway, such as interferon-γ (IFN-γ) and common γ chain cytokines, contribute to the destruction of insulin-secreting β cells by CD8^+^ T cells in type 1 diabetes (T1D). We previously showed that JAK1/JAK2 inhibitors reversed autoimmune insulitis in non-obese diabetic (NOD) mice and also blocked IFN-γ mediated MHC class I upregulation on β cells. Blocking interferons on their own does not prevent diabetes in knockout NOD mice, so we tested whether JAK inhibitor action on signaling downstream of common γ chain cytokines, including IL-2, IL-7 IL-15, and IL-21, may also affect the progression of diabetes in NOD mice. Common γ chain cytokines activate JAK1 and JAK3 to regulate T cell proliferation. We used a JAK1-selective inhibitor, ABT 317, to better understand the specific role of JAK1 signaling in autoimmune diabetes. ABT 317 reduced IL-21, IL-2, IL-15 and IL-7 signaling in T cells and IFN-γ signaling in β cells, but ABT 317 did not affect GM-CSF signaling in granulocytes. When given *in vivo* to NOD mice, ABT 317 reduced CD8^+^ T cell proliferation as well as the number of KLRG^+^ effector and CD44^hi^CD62L^lo^ effector memory CD8^+^ T cells in spleen. ABT 317 also prevented MHC class I upregulation on β cells. Newly diagnosed diabetes was reversed in 94% NOD mice treated twice daily with ABT 317 while still on treatment at 40 days and 44% remained normoglycemic after a further 60 days from discontinuing the drug. Our results indicate that ABT 317 blocks common γ chain cytokines in lymphocytes and interferons in lymphocytes and β cells and are thus more effective against diabetes pathogenesis than IFN-γ receptor deficiency alone. Our studies suggest use of this class of drug for the treatment of type 1 diabetes.

## Introduction

Type 1 diabetes (T1D) is an autoimmune disease characterized by hyperglycemia due to destruction of insulin-secreting β cells primarily by the contents of cytotoxic granules of CD8^+^ T cells (1). Many cytokines are important for the development of T1D, acting on both the immune system and the target β cells (2). Two cytokine families that are important in diabetes pathogenesis are interferons (IFN), including type 1 IFNs and IFN-γ, and the common γ chain cytokines, including IL-2, IL-4, IL-7, IL-9, IL-15, and IL-21. IFNs increase MHC class I expression on β cells, which allows activated CD8^+^ T cells to recognize β cells and kill them directly in both NOD mice and humans (3–5). IFNs are also required for upregulation of chemo-attractants and adhesion molecules in islets to enable infiltration of T cells (6). Common γ chain family cytokines have critical roles in T cell development, proliferation, survival, and differentiation. Investigations show that IL-2 and IL-21 are the prime cytokines needed for growth and differentiation of CTL (7, 8). IL-7 and IL-15 are responsible for the generation of self-reactive T-cells in autoimmune diseases (9). Treatment with anti-IL-7 receptor-α reverses established autoimmune diabetes in NOD mice (10). Similarly, IL-21 is critical for the development of T1D in NOD mice and NOD mice deficient in IL-21 receptors were protected from diabetes (11, 12).

Both interferons and the common γ chain cytokines signal through the JAK-STAT (signal transducer and activator of transcription) pathway. Our lab previously demonstrated that the JAK1/JAK2 inhibitor AZD1480 blocked IFN-γ mediated MHC class I upregulation on β cells, reduced immune cell infiltration into pancreatic islets, and reversed established autoimmune insulitis in NOD mice (13). However, the genetic absence of IFN-γ or its receptor does not prevent either insulitis or diabetes in NOD mice (14–16), nor does blocking IFN-γ receptors only on β cells in NOD mice (17). While we know from our previous work that JAK inhibitors block IFN-γ signaling *in vivo*, it is also likely that they affect the progression of diabetes in NOD mice by blocking common γ chain cytokine signaling to inhibit T cell proliferation and survival.

JAK inhibitors have been investigated for autoimmune and inflammatory diseases because they block many cytokines that function in the immune system. Currently, three JAK inhibitors, baricitinib, tofacitinib, and upadacitinib, have been approved for clinical use for rheumatoid arthritis and ruxolitinib for myelofibrosis. The JAK1 selective inhibitor upadacitinib (ABT-494), has 74- and 58-fold greater selectivity for JAK1 over JAK2 and JAK3, respectively (18, 19). Because JAK2 signals downstream of GM-CSF, EPO, and TPO, JAK1 selective inhibitors are expected to have a better safety profile than JAK1/JAK2 inhibitors with a lower risk of anemia (20).

In the present study, we used ABT 317, a JAK1-selective inhibitor, at exposures that are pharmacologically relevant to better understand the mechanism by which JAK inhibitors reverse autoimmune diabetes in NOD mice. The effect of ABT 317 on the function of common γ chain cytokines and T cell proliferation as well as the possible sides effects of ABT 317 on blood were characterized.

## Materials and Methods

### Mice

NOD/Lt and C57BL/6 mice were bred and maintained at St. Vincent’s Institute. All animal studies were approved by the institutional animal ethics committee.

### JAK1 Selective Inhibitor

The JAK1 selective inhibitor ABT 317 was provided by AbbVie Bioresearch Center (Worcester, MA, USA). In cells (21, 22), ABT 317 inhibited IL-6-stimulated pSTAT3 (TF-1 cells, IC_50_ of 0.016 µM) and IL-2-stimulated pSTAT5 (T-blasts, IC_50_ of 0.030 µM). In contrast, ABT 317 showed reduced potency against cytokine signaling pathways mediated by JAK2 (EPO-stimulated pSTAT5 in UT-7 cells, IC_50_ of 0.50 µM) or TYK2 (IL-12-stimulated pSTAT4 in T-blasts, IC_50_ of 0.25 µM). These cellular potency data (**Table S1**) are consistent with JAK1 selectivity of ABT 317.

For use *in vitro*, ABT 317 was resuspended at 10 mM in DMSO and diluted in media to a final concentration of 2 µM. Splenocytes and whole blood cells were incubated with 2 µM ABT 317 for 1 h at 37°C. For use *in vivo*, 3 mg/kg or 10 mg/kg of drug was delivered twice daily by oral gavage in the vehicle 0.5% hydroxypropyl methylcellulose in sterile H_2_O. The JAK1/JAK2 inhibitor baricitinib was purchased from SelleckChem and used at a concentration of 3 mg/kg *in vivo* made up in a vehicle of 0.5% methyl cellulose.

For pharmacokinetics, non-fasted male NOD mice were divided into three groups (n = 2/group) for 3, 10, or 30 mg/kg oral gavage of ABT 317. Plasma was collected *via* orbital bleed at 0.5, 1, 3, 7, 12, and 24 h after gavage and analyzed for concentration of ABT 317.

### Preparation of Single-Cell Suspensions

To isolate pancreatic islets, the bile duct was injected with 1.3 U/ml collagenase P (Roche, Basel, Switzerland) in complete HBSS (Lonza), removed and digested for 17 min at 37°C in a water bath, followed by vigorous shaking for 1 min. The digested tissue was filtered through a 500 μm mesh, then washed with RPMI (Invitrogen). Islets were purified using a histopaque-1077 density gradient (Sigma-Aldrich) and dispersed into single cells using bovine trypsin (342 U/ml) and 2 mM EDTA in PBS. Finally, the cells were washed and rested in complete RPMI for at least 1 h at 37°C.

Single-cell suspensions from the spleens were prepared by mechanical tissue disruption through a 70 μm cell strainer followed by red cell lysis. Cells were resuspended in FACS buffer (0.5% FCS in PBS) and filtered through a nylon mesh.

### Stimulation of Islets, Splenocytes, and Peripheral Blood

Whole islets were treated with vehicle or ABT 317 for 1 h before culture with 10 ng/ml IFN-γ for 72 h, and analysis by flow cytometry. Splenocytes and whole blood were stimulated with 100 U/ml IL-2, 50 ng/ml IL-7, 30 ng/ml IL-15, 25 ng/ml IL-21, or 40 ng/ml GM-CSF for 30 min at 37°C. Red blood cells were lysed (155 mmol/L NH_4_Cl, 10 mmol/L Tris-HCl, pH 7.5). Leukocytes were analyzed by flow cytometry.

### Injection of BrdU

Bromo-deoxyuridine (BrdU) was diluted to 10 mg/ml in sterile PBS. Mice were injected i.p. with 200 µl (2 mg) of BrdU solution. Mice were harvested 24 h after injection.

### Flow Cytometry

Mouse islet cells were stained with anti-CD45 and biotinylated anti-H2Kd (BD Pharmingen) followed by streptavidin-allophycocyanin (BioLegend). β‐cells were identified by autofluorescence (23). Dead cells were excluded using propidium iodide. The immune cells infiltrating islets and spleens were stained with anti-CD45, anti-CD3, anti-CD4, anti-CD8a, anti-CD44, anti-CD62L, anti-KLRG-1, anti-CD11b, anti-Ly6G (all BioLegend), anti-BrdU, anti-pSTAT3, and anti-pSTAT5 (all BD Bioscience). Data were collected on the FACS Fortessa cell analyzer (BD Bioscience, San Jose, CA, USA) and were analyzed using FlowJo Software version 10 (TreeStar, Inc, Ashland, OR, USA).

### Histology

Pancreases were Bouins fixed and paraffin embedded, 5 µm sections were cut from three levels (200 µm apart) and stained with hematoxylin and eosin (H&E). Insulitis was scored using the following scale: 0 = no infiltrate, 1 = peri-islet infiltrate, 2 = extensive (>50%) peri-islet infiltrate, 3 = intraislet infiltrate, and 4 = extensive intraislet infiltrate (>80%) or total β cells loss. The percentage of islets with each score per pancreas was calculated by addition of the scores for the three sections.

### Western Blot

Spleen cells were cultured with 2 µM ABT 317 for 1 h prior to the addition of 25 ng/ml IL-21 for 15 min. Western blot for pSTAT3 and actin were performed using standard procedures.

### *In Vivo* Treatment of Mice

For anti-CD3 monoclonal antibody (mAb) treatment, NOD mice were treated with ABT 317 or vehicle by oral gavage for 2 days. On day 2, mice were injected intraperitoneally with 10 µg anti-mouse CD3 Ab (clone 145–2C11; Bio X Cell, West Lebanon, NH, USA). Islets were isolated on day 3 and analyzed by flow cytometry.

### Analysis of Diabetes

Mice were monitored for diabetes by measurement of blood glucose levels with Advantage II Glucose Strips (Roche) twice a week. Mice suspected of hyperglycemia were tested on the following day, and two consecutive blood glucose readings >13.9 mmol/L was considered diabetic.

When female NOD mice were diagnosed with diabetes, they were implanted with a LinBit insulin pellet (LinShin Canada, Inc.) the following day (24). At the same time, mice were treated with ABT 317 or vehicle twice daily by oral gavage for 40 days after a diabetes diagnosis. Mice were monitored twice weekly throughout the study for blood glucose.

### Statistical Analysis

Statistical analysis was performed using GraphPad Prism 8 Software (GraphPad, San Diego, CA, USA). All data shown as bar graphs are represented as the mean ± SEM. A p value of <0.05 was considered to be significant.

## Results

### Pharmacokinetics of the JAK1 Selective Inhibitor ABT 317 in NOD Mice

The pharmacokinetic (PK) profile of ABT 317 was assessed in NOD mice to confirm that sufficient drug exposure could be achieved and that the corresponding drug level was sufficient to test the JAK1 selectivity. The pharmacokinetic behavior of ABT 317 in NOD mice was evaluated following escalating single oral doses. The compound exhibited rapid absorption, with plasma concentrations reaching their mean maximum concentration between 0.5 and 1 h. The exposure increased less than dose proportionally between 3 and 30 mg/kg, and the mean elimination half-life ranged from 1.9 to 2.6 h (**Figure S1** and **Table 1**). The levels of ABT 317 at the 3 and 10 mg/kg doses informed dose selection as well as dosing frequency for the subsequent efficacy studies.

**Table 1:** Summary of pharmacokinetic parameters for ABT 317 in NOD mice.

	T_1/2_	C_max_	AUC
3 mg/kg, oral	2.6°	40.3	150
10 mg/kg, oral	2.1°	74.7	302
30 mg/kg, oral	1.9°	191	1040

harmonic mean; t_1/2_ [h]; C_max_ [ng/ml]; AUC [ng*h/ml]. N = 2 mice per time point.

### ABT 317 Blocks IFN-γ Signaling and Common γ Chain Cytokines IL-2, IL-7, IL-15, and IL-21 *In Vitro*

**Figure 1:**
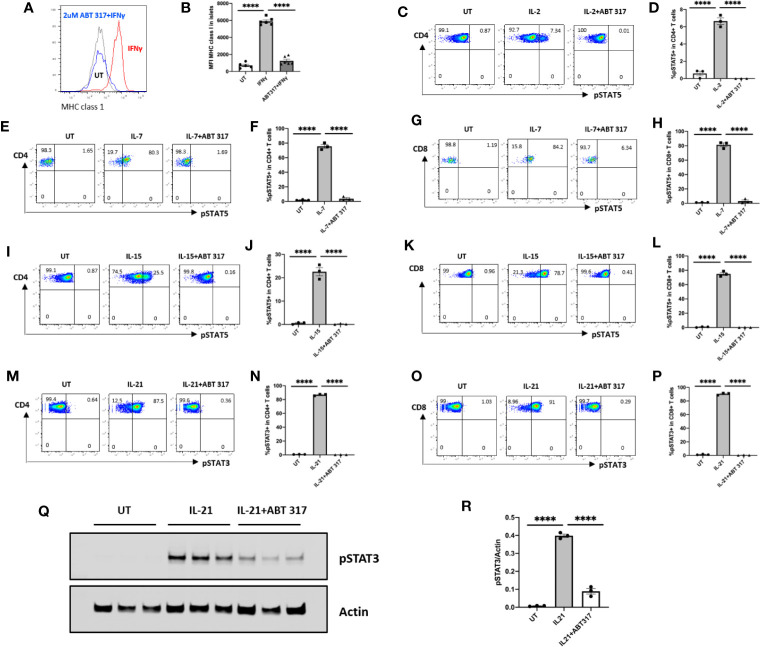
ABT 317 blocks IFN-γ and common γ chain cytokine signaling *in vitro.* **(A, B)**: The mean fluorescence intensity (MFI) of MHC class 1 expression on mouse islet cells after 48 h of treatment with IFN-γ in the presence of vehicle or 2 µM ABT 317. Individual mice (n = 5–6) from three independent experiments are shown with the mean ± SEM. **C–P**: Spleen cells were cultured with or without 1 µM ABT 317 for 1 h prior to the addition of cytokine for 15 min to analyze the percentage of: **(C, D)**: IL-2-induced pSTAT5 positive CD4^+^ T cells. **(E–H)**: IL-7 induced pSTAT5 positive CD4^+^ T cells **(E, F)** and CD8^+^ T cells **(G, H)**. **I–L**: IL-15 induced pSTAT5 positive CD4+ T cells **(I, J)** and CD8+ T cells **(K, L)**. **M–P**: IL-21 induced pSTAT3 positive CD4+ T cells (**I** and **J**) and CD8+ T cells **(K, L)**. **(Q, R)**: Western blot of pSTAT3 and Actin for spleen cells cultured with 2 µM ABT 317 for 1 h prior to the addition of 25 ng/ml IL-21 for 15 min. Data show a **(Q)** representative blot and **(R)** individual mice (n = 3) from one experiment with mean ± SEM. Statistical significance: ****P < 0.0001, one-way ANOVA with Tukey’s test for multiple comparisons.

Isolated mouse islets were treated with ABT 317 or vehicle, then stimulated with IFN-γ. Flow cytometry data indicates that ABT 317 prevented IFN-γ-induced upregulation of MHC class 1 on β cells in mice *in vitro* (**Figures 1A, B**).

Isolated mouse splenocytes were treated with ABT 317 or vehicle for 1 h, then stimulated for 15 min with common γ chain cytokines IL-2, IL-7, IL-15, or IL-21 separately. IL-2, IL-7, or IL-15-induced phosphorylation of STAT5 in T cells was inhibited by ABT 317 (**Figures 1C–L** and **Figures S2A–F**). ABT 317 also blocked IL-21-induced phosphorylation of STAT3 in T cells (**Figures 1M–P** and **Figures S2G–J**). These data were confirmed by western blot analysis (**Figures 1Q, R**). The effect of JAK inhibitor on blocking IL-21 signaling through pSTAT3 was dose dependent and similar to other JAK inhibitors baricitinib and tofacitinib (**Figures S2K–L**). These data indicate that ABT 317 can block cytokine signaling in both β cells (IFNγ) and T cells (IL-2, IL-7, IL-15, and IL-21).

### ABT 317 Reduces Proinflammatory Cytokine Signaling in Blood T Cells and β Cells

**Figure 2:**
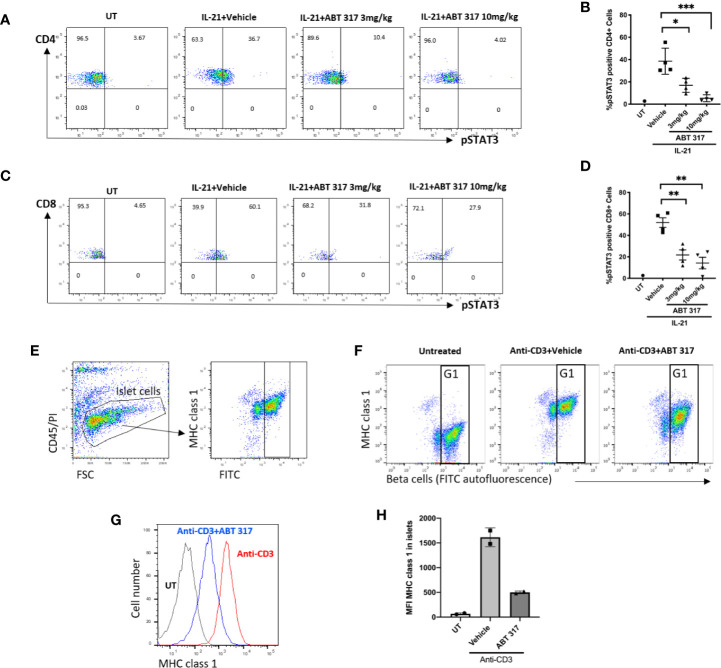
ABT 317 reduces proinflammatory cytokine signaling in blood T cells and in β cells. **(A–D)**: Peripheral blood was collected from 5–6-week old NOD mice treated with vehicle, 3 mg/kg ABT 317 or 10 mg/kg ABT 317 for 3 days, stimulated *ex vivo* with IL-21, and analyzed by FACS for pSTAT3 positive CD4^+^ T cells **(A, B)** and pSTAT3 positive CD8^+^ T cells **(C, D)**. Data show representative dot plots and pooled data from n = 4 individual mice/time point and treatment group from two independent experiments. Individual mice are shown with the mean ± SEM. Statistical significance: **(B)**: *P = 0.0162, ***P < 0.005; **(D)**: **P = 0.0080 between vehicle and 3mg/kg ABT 317, **P = 0.0018 between vehicle and 10 mg/kg, one-way ANOVA with Tukey test for multiple comparisons. **(E)**: FACS gating process of beta cells is shown. **(F–H)**: MHC class I expression on β cells isolated from 10- to 12-week old C57BL/6 mice treated with or without anti-CD3 and vehicle or 10 mg/kg ABT 317. β cells are identified as CD45^-^PI^-^ (live cells) with high autofluorescence (G1). Individual mice (n = 2) from one experiment is shown with the mean ± SD.

To confirm that ABT 317 blocked cytokine signaling *in vivo*, 5-6-week old NOD mice were treated with 3 mg/kg ABT 317 or 10 mg/kg ABT 317 or vehicle twice daily by oral gavage for 3 days. Whole blood was stimulated with IL-21 for 15 min *ex-vivo* followed by flow cytometry. Both 3 and 10 mg/kg ABT 317 inhibited IL-21-induced STAT3 phosphorylation in T cells (**Figures 2A–D** and **Figure S3**).

We used a rapid non-autoimmune model to test whether ABT 317 could inhibit cytokine-induced MHC class I upregulation on β cells *in vivo*. Injecting anti-CD3 mAbs into mice induces systemic secretion of interferons and the upregulation of MHC class I on β cells (13). C57BL/6 mice were treated with 10 mg/kg ABT 317 or vehicle twice daily by oral gavage for 2 days, and on the second day mice were injected with 10 µg of anti-CD3 mAb. Islets were isolated for flow cytometry the next day. Mice that received the vehicle and anti-CD3 mAbs had increased MHC class 1 compared to untreated mice (**Figures 2E–H**), while MHC class 1 was significantly reduced in mice receiving a combination of ABT 317 and anti-CD3 mAbs. Thus, ABT 317 blocks proinflammatory cytokine signaling in T cells and β cells *in vivo*.

### ABT 317 Does Not Affect GM-CSF Signaling in Granulocytes

Since JAK2 is downstream of erythropoietin (EPO), granulocyte-macrophage colony-stimulating factor (GM-CSF), and thrombopoietin (TPO), neutropenia has been found in clinical trials with the JAK inhibitors baricitinib (25, 26) and tofacitinib (27, 28) both of which target JAK2 to some extent. In theory, a JAK1-selective inhibitor should have a fewer side effects on the blood system because it does not affect cytokines dependent on the JAK2 signaling pathway (29, 30).

**Figure 3:**
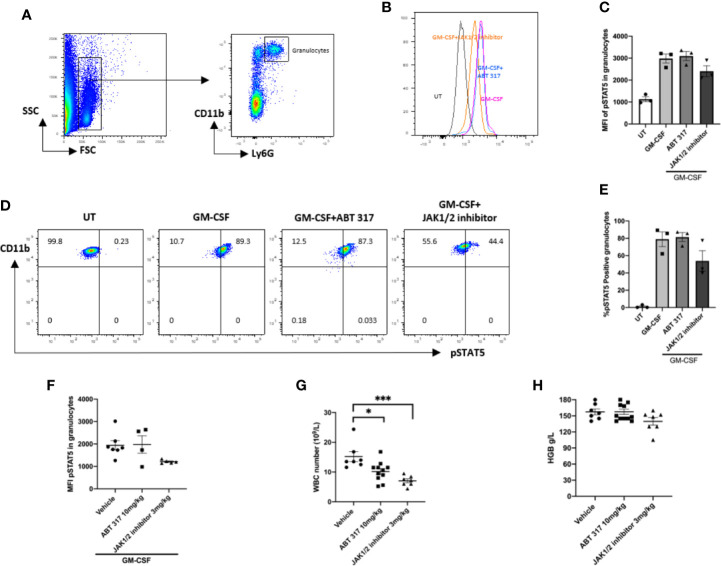
ABT 317 does not affect GM-CSF signaling or blood cell counts. **(A)** FACS gating process of granulocytes is shown. (**B–E**) Peripheral blood was cultured with or without 2 µM ABT 317 or 1 µM JAK1/JAK2 inhibitor for 1 h prior to the addition of GM-CSF for 30 min to analyze the mean fluorescence intensity (MFI) expression of phosphorylated STAT5 in granulocytes **(B, C)** and the percentage of pSTAT5 positive granulocytes **(D, E)**. Data show individual mice (n = 3) and mean ± SEM for three independent experiments. **(D)** Representative plots are shown. **(F)** Peripheral blood was collected from 6–8-week-old NOD mice treated with vehicle, 10 mg/kg ABT 317 or 3 mg/kg JAK1/JAK2 inhibitor for 3 days and stimulated *ex vivo* with GM-CSF. The MFI of phosphorylated STAT5 in granulocytes is shown for n = 5–7 individual mice from two independent experiments. **(G, H)** Peripheral blood was collected from NOD mice treated with vehicle, 10 mg/kg ABT 317 or 3 mg/kg JAK1/JAK2 inhibitor for 3 weeks. **(G)** White blood cell counts and **(H)** hemoglobin levels are shown for n = 7–10 individual mice from three independent experiments. Data are shown as representative plots and the mean ± SEM of pooled data. Statistical significance: *P = 0.0365, ***P = 0.0004, one-way ANOVA with Tukey’s test for multiple comparisons.

*In vitro*, ABT 317 did not affect GM-CSF signaling in granulocytes (**Figures 3A–E**). When given *in vivo* orally to NOD mice daily for 3 days, ABT 317 did not block GM-CSF signaling in granulocytes (**Figure 3F**). After 3 weeks of treatment with 10 mg/kg ABT 317, there was a significant decrease in the number of white blood cells after ABT 317 treatment (**Figure 3G**) suggesting that the higher dose of ABT 317 may affect JAK3 signaling. Hemoglobin was not significantly reduced (**Figure 3H**).

### ABT 317 Reduces Immune Cell Infiltration and T-Cell Proliferation in Islets

**Figure 4:**
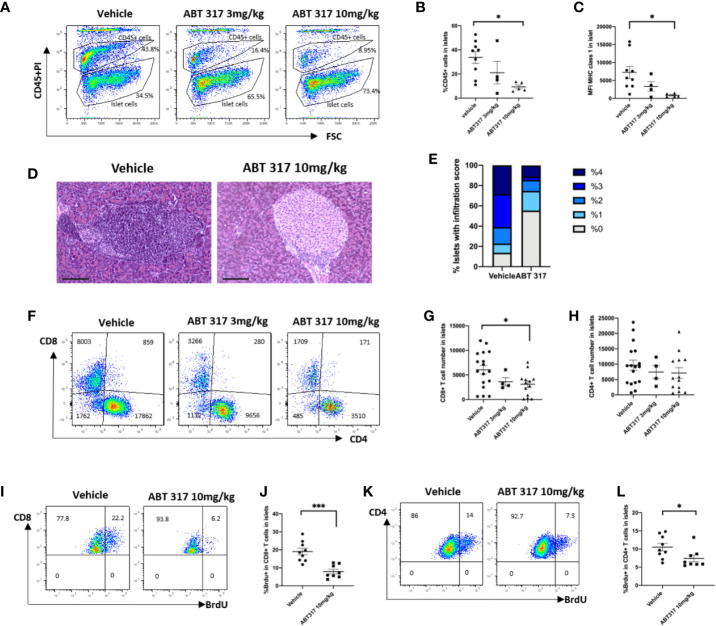
ABT 317 reduces immune cell infiltration and T-cell proliferation in islets. **(A, B)**: The percentage of CD45^+^ cells in islets from 13-week-old NOD mice treated with vehicle or 3 mg/kg or 10 mg/kg ABT 317 for 3 weeks. **(A)**: Representative plots showing gating for CD45^+^ and CD45^-^ islet cells. **C**: MFI of MHC class I expression on the β cell surface from NOD mice treated with vehicle or 3 or 10 mg/kg ABT 317 for 3 weeks. Data for **(A–C)** are n = 9 mice combined from two experiments (four to five mice per group in each experiment). **(D–F)**: CD45^+^CD3^+^ live T cells in islets from 13-week-old NOD mice treated with vehicle or 3 or 10 mg/kg ABT 317 for 3 weeks. **(D, E)**: Insulitis in pancreas harvested from 10 mg/kg ABT 317 (n = 3) or vehicle (n = 8) treated 150-day old female NOD mice. **(D)** Representative H&E stained sections, magnification 100×, scale bar 100 µm. **(E)** Pooled insulitis scores. P < 0.0001, two-way ANOVA. **(F)**: Representative flow cytometry dot plots. **(G, H)**: The numbers of CD8^+^ T cells **(G)** and CD4^+^ T cells **(H)**. Data are n = 13 mice combined from three experiments (four or five mice per group in each experiment). Data are shown as the mean ± SEM. Statistical significance: *P < 0.05, One-way ANOVA with multiple comparisons. **(I–L)**: The percentage of BrdU positive CD45^+^CD3^+^ T cells in islets from 13-week-old NOD mice treated with vehicle or 10 mg/kg ABT 317 for 3 weeks. **(I, K)**: Representative flow cytometry dot plots. **(J, K)**: The percentage of BrdU positive CD8^+^ T cells **(J)** and the percentage of BrdU positive CD4^+^ T cells **(L)**. Data are n = 8–9 mice with the mean ± SEM for two independent experiments. Statistical significance: *P < 0.05, ***P = 0.0002, Student t test.

When given to pre-diabetic NOD mice, 10 mg/kg ABT 317 significantly reduced MHC class 1 expression on the β cell surface and reduced the proportion of CD45^+^ cells in islets (**Figures 4A–C**). The expression of MHC class I and proportion of CD45^+^ cells in islets was variable in the vehicle-treated mice and in mice treated with 3 mg/kg ABT 317 and was not significantly different. The majority of islets in the vehicle-treated NOD mice had extensive immune cell infiltration and β cell destruction. In contrast ABT 317-treated mice were significantly protected from developing insulitis (**Figures 4D, E**). Then we tested whether ABT 317 treatment affected the number of T cells in islets. While the number of CD4^+^ T cells was not affected, the number of CD8^+^ T cells in islets was significantly reduced after treatment with 10 mg/kg ABT 317 (p < 0.05) (**Figures 4F–H**).

We then used BrdU incorporation to test whether ABT 317 affects T-cell proliferation. Proliferation of CD8^+^ and CD4^+^ T cells in islets was significantly reduced after treatment with ABT 317 (**Figures 4I–L**).

### ABT 317 Reduces Effector Memory CD8^+^ T Cells *In Vivo*

**Figure 5:**
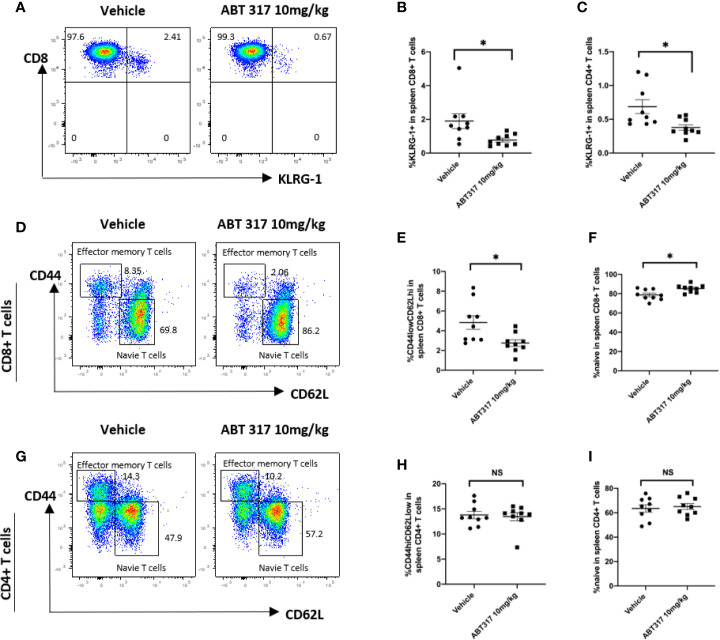
ABT 317 reduces the proportion of effector and memory CD8^+^ T cells *in vivo.* **(A–C)**: The percentage of KLRG1^+^ T cells in spleen from 13-week-old NOD mice treated with vehicle or 10 mg/kg ABT 317 for 3 weeks. **(A)** Representative flow cytometry plot and **(B)** pooled data from n = 9 individual mice for KLRG1^+^CD8^+^ T cells. **(C)** Pooled data from n = 9 individual mice for KLRG1^+^CD4^+^ T cells. **(D–F)**: **(D)** Representative flow cytometry plots, **(E)** the percentage of CD44^hi^CD62L^lo^CD8^+^ T cells and **(F)** the percentage of CD44^lo^CD62L^hi^CD8^+^ T cells in the spleen from 15–16-week-old NOD mice treated with vehicle or 10 mg/kg ABT 317 for 3 weeks. **(G–I)**: **(G)** Representative flow cytometry plots, **(H)** the percentage of CD44^hi^CD62L^lo^CD4^+^ T cells and **(I)** the percentage of CD44^lo^CD62L^hi^CD4^+^ T cells in the spleen from 13-week-old NOD mice treated with vehicle or 10 mg/kg ABT 317 for 3 weeks. Pooled data are from n = 9 individual mice with the mean ± SEM. Statistical significance: **(B)**: *P = 0.0234, **(C)**: *P = 0.0140, **(E)**: *P = 0.0156, **(F)**: *P = 0.0106, NS, not significant. Student t test.

Common γ chain cytokines control effector and memory T-cell development and survival (31) and prevent the death of activated T cells (32). KLRG1 is a marker of effector T cells. Treatment of NOD mice with 10 mg/kg ABT 317 significantly reduced the percentage of both KLRG1^+^CD8^+^ T cells and KLRG1^+^CD4^+^ T cells in the spleen (**Figures 5A–C**).

The proportion of CD44^hi^CD62L^lo^ effector memory CD8^+^ T cells in the spleen was significantly reduced after 10 mg/kg ABT 317 treatment (**Figures 5D, E**), with a corresponding increase in the proportion of naïve CD44^lo^CD62L^hi^CD8^+^ T cells (**Figure 5F**). However, ABT 317 treatment did not affect the percentage of effector memory (**Figures 5G, H**) or naïve CD4^+^ T cells (**Figure 5I**).

### ABT 317 Reverses Newly Diagnosed Diabetes in NOD Mice

**Figure 6:**
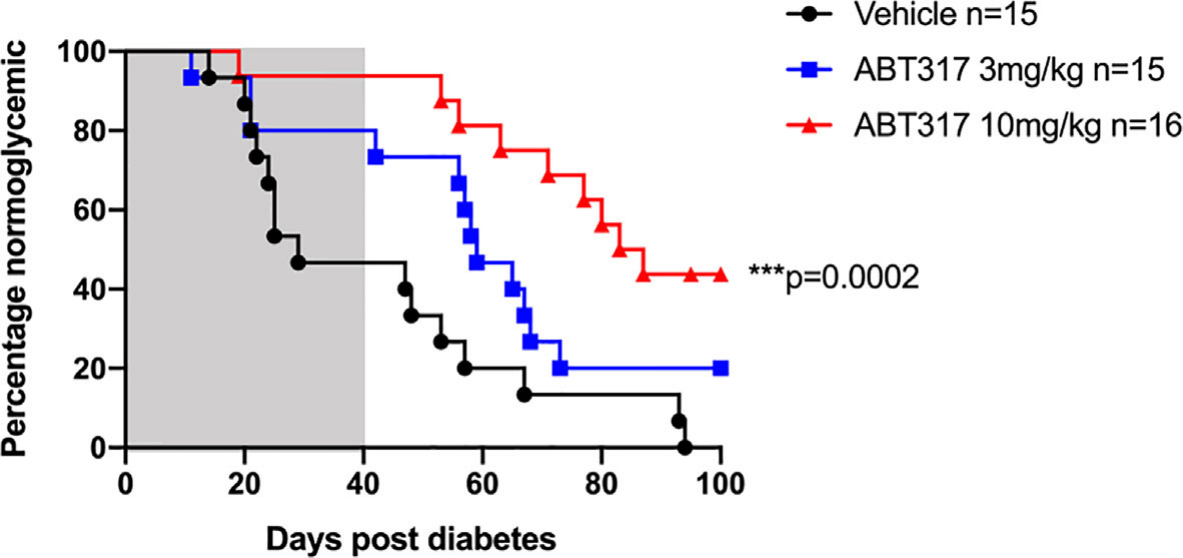
ABT 317 reverses newly diagnosed diabetes in NOD mice. Blood glucose levels were monitored in NOD mice treated with a slow release insulin pellet and randomized to receive vehicle (n = 15), 3 mg/kg ABT 317 (n = 15), or 10 mg/kg ABT 317 (n = 16) twice daily for 40 days after diagnosis of hyperglycemia. Survival curve analysis of the percentage of normoglycemic mice. ***P = 0.0002, log-rank test. Shaded area represents treatment period of 40 days.

We treated diabetic female NOD mice with a slow-release insulin pellet and twice daily gavage of 3 or 10 mg/kg ABT 317 or vehicle for 40 days. Over half vehicle-treated mice became hyperglycemic again around 2–3 weeks, which was after the effects of the insulin pellet wore off. However, 73.3% of 3 mg/kg ABT 317-treated mice and 93.75% of 10 mg/kg ABT 317-treated mice remained normoglycemic for the treatment period of 40 days (**Figure 6**). After treatment ceased, ABT 317-treated mice remained normoglycemic for another 10 days. At 60 days after the original diabetes diagnosis, half of the 3 mg/kg ABT 317-treated mice and 75% of the 10 mg/kg ABT 317-treated mice remained normoglycemic. At 100 days after the original diabetes diagnosis, all of vehicle treated mice remained diabetic, but 20% of the 3 mg/kg ABT 317-treated mice reversed diabetes and 43.75% of the 10 mg/kg ABT 317-treated mice reversed diabetes, indicating prolonged effects of ABT 317 after the treatment ceased.

## Discussion

We conducted pre-clinical analysis of the JAK1-selective inhibitor, ABT 317, in NOD mice to determine its efficacy in T1D, and to better understand the mechanism by which JAK inhibitors prevent and reverse autoimmune diabetes. The results show that ABT 317 acts on both β cells and autoreactive T cells by inhibiting IFN-γ and common γ chain cytokine signaling, making β cells less easily recognized by CD8^+^ T cells by preventing IFN-γ mediated MHC class 1 upregulation on β cells and blocking proliferation of effector memory CD8^+^ T cells by blocking common γ chain cytokine signaling. Our expectation was that maintaining exposures in mice that would result in complete JAK1 inhibition without significant inhibition of JAK2 would result in efficacy without significant impact on blood cells. ABT 317 did not affect GM-CSF signaling in granulocytes and did not result in a change in hemoglobin levels in NOD mice.

In our previous studies, we showed that a JAK1/JAK2 inhibitor, at doses that would fully inhibit both JAK1 and JAK2, prevented IFNγ-mediated upregulation of MHC class 1 expression on β cells and reduced the formation of immunological synapses between CD8^+^ T cells and β cells thus preventing β cell destruction (13). NOD mice were protected from diabetes after JAK inhibitor treatment. However, there is evidence that blocking IFN-γ signaling in NOD mice does not prevent diabetes. Therefore, we set out to test whether the effect of ABT 317 on common γ chain cytokine signaling could explain the difference between effects of IFN-γ/IFNγR deficiency and JAK1 inhibition on diabetes in NOD mice. Common γ chain cytokines are essential for T cell development and especially effector memory T-cell generation. Conversely, the availability of cloned CTL from IFN-γ-deficient mice with *in vitro* and *in vivo* functional activities in influenza infection indicates that CD8^+^ cytotoxic T cells can develop normally in the absence of IFN-γ signaling (14, 33).

In the NOD mouse model of T1D, we found that ABT 317 inhibited CD8^+^ T-cell proliferation *in vivo*, while there was less effect on CD4^+^ T-cell proliferation. The pathology of T1D in both NOD mice and humans is marked by the presence of autoreactive cytotoxic CD8^+^ T lymphocytes (CTLs) that mediate β cell destruction. These cells acquire effector function in the islets, a process dependent on cytokine action (34, 35). Common γ chain cytokines are important for T-cell proliferation and the generation of self-reactive T cells in autoimmune diseases (9). JAK inhibitors block the catalytic activity of JAKs and they are unlikely to affect cells that are not responding to cytokine stimulation, for example in a non-inflammatory setting (36). Therefore, we propose that ABT 317 acts by inhibiting common γ chain cytokine-dependent proliferation of CD8+ T cells in the islets, instead of CD4^+^ T-cell proliferation. Additionally, ABT 317 also reduced effector and effector memory CD8^+^ T cells in the periphery. Our results show that ABT 317 decreased the percentage of both KLRG1^+^CD4^+^ and KLRG1^+^CD8^+^ effector T cells significantly (**Figure 5**). In CD8^+^ T cells, KLRG1 expression correlates with cytotoxicity (37) and it is upregulated on cytotoxic T cells in the islets of NOD mice (35, 38). Furthermore, in NOD mice, IGRP antigen-specific CD8^+^ T cells that have migrated from the islets into the periphery express elevated levels of KLRG1 compared to other CD8^+^ T cells (38). This inhibition of CD8^+^ T-cell proliferation resulted in a prolonged effect of the ABT 317 on blood glucose level control. The effects of common γ chain cytokines on the expansion of memory T cell populations is also widely studied. It was shown that memory CD8^+^ cells but not CD4^+^ T cells rely on IL-7 or IL-15 to undergo homeostatic proliferation (39, 40). Consistent with this, both IL-15- and IL-15R–deficient mice have reduced numbers of memory CD8^+^ T cells in spleens and LNs (41, 42). However, it remains unclear if ABT 317 only affects proliferation of T cells or if it also results in reduced survival of T cells, presumably due to lack of growth factor signaling (31). Comparison of intracellular STAT3 or STAT5 phosphorylation, proliferation markers, as well as apoptosis markers on memory T cells would be needed to reveal this.

The cytokines tested in this study, IL-2, IL-7, IL-15, and IL-21, are known to phosphorylate JAK1 and JAK3 and activate pSTAT3 and pSTAT5. IL-2, IL-7, IL-9, and IL-15 mainly activate STAT5 (STAT5A and STAT5B) (43), while IL-21 mainly activates STAT3 (44), which is consistent with our *in vitro* results. ABT 317 blocked IL-2, IL-7, and IL-15 mediated phosphorylation of STAT5 and IL-21 mediated phosphorylation of STAT3 (**Figure 1**).

Although common γ chain cytokines function through JAK1 and JAK3, a dominant role of JAK1 over JAK3 has been reported. It was shown that to maintain IL-7 signaling, continuous synthesis of new JAK1 protein is required (45). In contrast, JAK3 catalytic activity is dispensable for IL-2 induced STAT5 phosphorylation (46). JAK3 supports signal transduction by phosphorylating JAK1, but JAK3 does not induce STAT5 phosphorylation directly. Therefore, JAK3 always cooperates with JAK1 for signaling, but specific inhibition of JAK3 is not sufficient to efficiently block common γ chain cytokine signal transduction required for strong immunosuppression (47). However, loss-of-function mutations in JAK3 in humans or experimental deletion of either one of these in mice results in severe combined immunodeficiency (47, 48). Tofacitinib, which targets JAK1 and JAK3, caused a dose-dependent, temporary decrease in NK cell count, and may affect the development and function of plasmablasts (49). Therefore, a JAK1 selective inhibitor in theory can inhibit common γ chain cytokine function while having better selectivity on JAK1 *versus* JAK3.

After the marketing of the first two pan-JAK inhibitors, tofacitinib and baricitinib, more recent research has been focused toward the development of more selective drugs with the ability to modulate the activity of only one JAK family member. JAK1 selective inhibitors were developed to improve the safety profile by minimizing the effects on JAK3 and JAK2. ABT 317 is an ATP competitive inhibitor selective for JAK1. Our findings indicate that ABT 317 is sufficient for preventing JAK1-dependent cytokine-dependent signal transduction. However, we saw a slight reduction in blood white blood cell numbers at 10 mg/kg ABT 317 compared to control, suggesting that high concentration ABT 317 may also inhibit cytokines other than those primarily dependent upon JAK1 at the concentration we used in NOD mice. Other research indicates that upadacitinib also inhibits the JAK2-dependent cytokines IL-3 and GM-CSF (30). The 10 mg/kg ABT 317 did not decrease hemoglobin in NOD mice, even after 3 weeks of treatment. Another JAK1-selective blocking agent filgotinib also did not result in anemia in rheumatoid arthritis patients (49). ABT 317 has not been used in clinical studies and JAK inhibitors have not yet been tested in T1D clinical trials. A reduction in albuminuria was noted in participants with diabetic kidney disease in a phase 2 trial of the JAK1/JAK2 inhibitor baricitinib (50). Available phase II and III data on upadacitinib and filgotinib confirm the efficacy observed in the first generation JAK inhibitors, baricitinib and tofacitinib, and indicate better safety profile compared to pan-JAK inhibitors’ effect on blood cells (19, 51–53).

In conclusion, we show that treatment of NOD mice with a JAK1 selective inhibitor, ABT 317, blocked MHC class 1 upregulation on β cells and inhibited common γ chain cytokine effects on T-cell proliferation and generation of effector and memory T cell to reverse autoimmune diabetes. Our research provides a basis for additional testing of JAK1 inhibitors to prevent immune-mediated β cell death in T1D.

## Data Availability Statement

The raw data supporting the conclusions of this article will be made available by the authors, without undue reservation.

## Ethics Statement

The animal study was reviewed and approved by St Vincent’s Hospital Animal Ethics Committee.

## Author Contributions

TG, GJ, SF, SA, SL, EP, TC, IV, and AL performed experiments, analyzed data, and revised the manuscript. TG, GJ, LO, BK, TK, and HT designed the study and wrote the manuscript. All authors contributed to the article and approved the submitted version.

## Funding

This work was funded by the Juvenile Diabetes Research Foundation (JDRF, SRA-2018-599-S-B), the National Health and Medical Research Council of Australia (GNT1126237 and GNT1150425) and fellowships from the JDRF and Manpei Suzuki Diabetes Foundation (SA). The St Vincent’s Institute receives support from the Operational Infrastructure Support Scheme of the Government of Victoria.

## Conflict of Interest

At the time of the study, AL and LO were both employees of Abbvie.

The remaining authors declare that the research was conducted in the absence of any commercial or financial relationships that could be construed as a potential conflict of interest.
