# Supplementary material for: The JAK1 Selective Inhibitor ABT 317 Blocks Signaling Through Interferon-γ and Common γ Chain Cytokine Receptors to Reverse Autoimmune Diabetes in NOD Mice
Source: Front Immunol. 2020 Dec 4;11:588543. doi: 10.3389/fimmu.2020.588543 (PMC7746546; doi:10.3389/fimmu.2020.588543)
Supplement: Supplementary file 1 [file DataSheet_1.docx]

**Supplementary table**

**Table S1 Cellular potency for ABT 317**

|  | JAK1 Cell  IL-6 stim pSTAT3 in TF-1 cells  (mM) | JAK2 Cell  Epo stim pSTAT5 in UT-7 cells  (mM) | JAK1,3 Cell  IL-2 stim pSTAT5 in T-blasts  (mM) | TYK2 Cell  IL-12 stim pSTAT4 in T-blasts  (mM) |
| --- | --- | --- | --- | --- |
| ABT 317 | 0.016 | 0.50 | 0.030 | 0.25 |

**Supplementary figures**


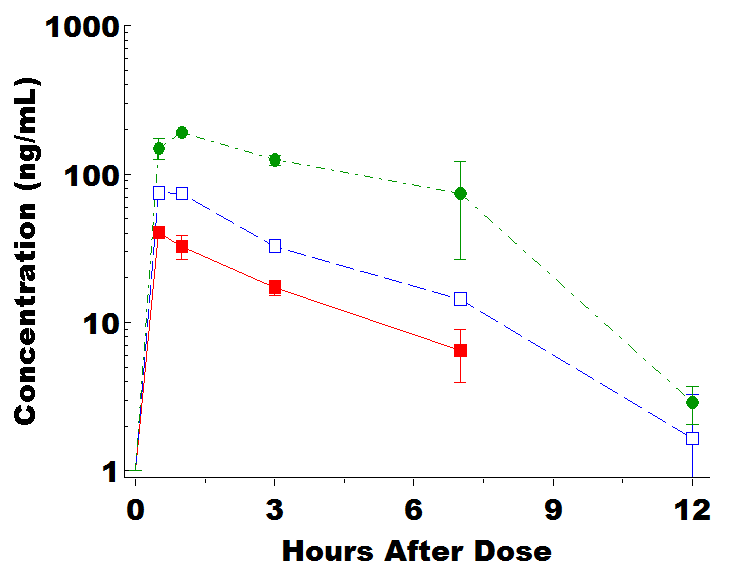

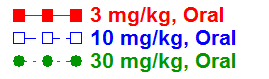


**Supplementary figure 1. Pharmacokinetics of ABT 317**

Mean (±SD) plasma concentrations of ABT 317 following 3, 10 or 30 mg/kg oral doses in male NOD mice, n=2 per time point.

**
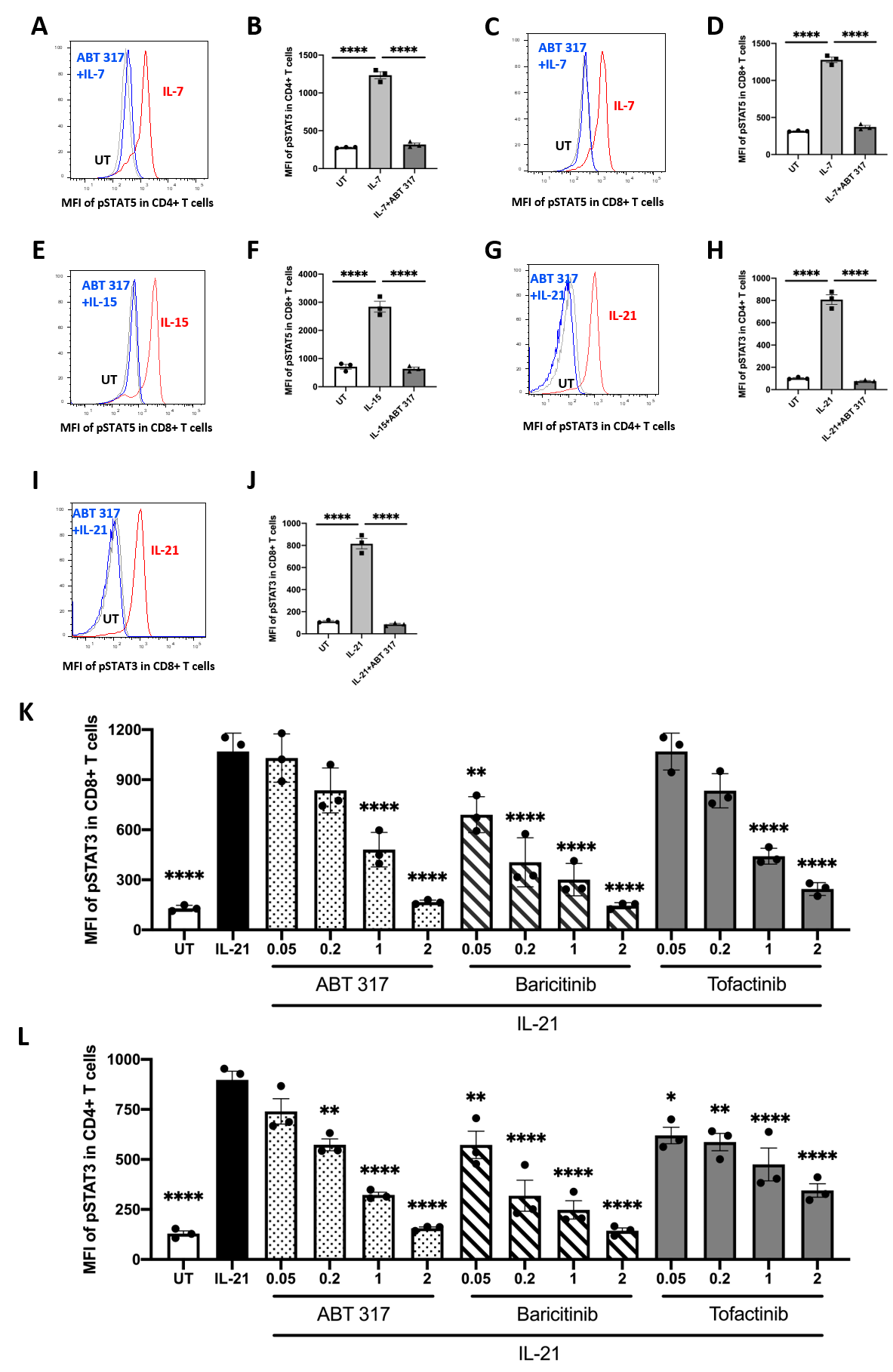
**

**Supplementary Figure 2. ABT 317 Blocks Common γ Chain Cytokine Signaling in Vitro**

A, B, C and D: Spleen cells were cultured with or without 1 µM ABT 317 for 1 hour prior to the addition of cytokine for 30 minutes. (A and B) The percentage of IL-7-induced phosphorylated STAT5 positive CD4^+^ T cells and (C and D) phosphorylated STAT5 positive CD8^+^ T cells. E and F: The percentage of IL-15-induced STAT5 positive CD8^+^ T cells. G, H, I and J: The percentage of IL-21-induced phosphorylated (G and H) STAT5 positive CD4^+^ T cells and (I and J) STAT5 positive CD8^+^ T cells. Data are shown as the mean ± SEM from n=3 individual mice. Statistical significance: ****P <0.0001, one-way ANOVA with Tukey’s test for multiple comparisons. K and L: MFI of pSTAT3 in CD8^+^ T cells (K) and CD4^+^ T cells (L) incubated with or without different doses (0.05 µM, 0.2 µM, 1 µM and 2 µM) of JAK inhibitors for 1 hour and then stimulated with 2.5ng/ml IL-21 for 30 minutes. Data show the mean ± SEM from n=3 individual mice, combined from 2 independent experiments. *p<0.05, **p<0.0005, ***p<0.001, ****p<0.0001, compared to IL-21 treatment, one-way ANOVA.

**
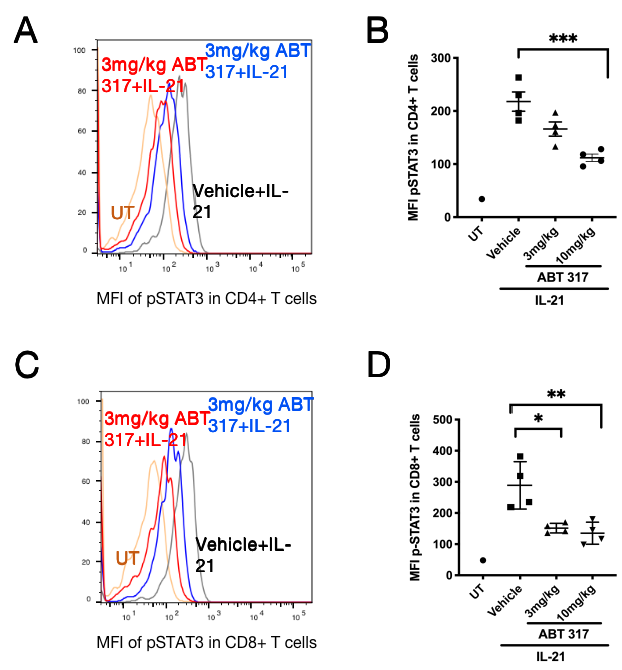
**

**Supplementary Figure 3: The JAK1 Selective Inhibitor ABT 317 Blocks Common γ Chain Cytokine Signaling in Vivo**

A, B, C and D: Peripheral blood was collected from 5-6-week old NOD mice treated with vehicle, 3 mg/kg or 10 mg/kg ABT 317 for 3 days, stimulated ex vivo with IL-21, and analyzed by FACS for (A and B) pSTAT3 in CD4^+^ T cells and (C and D) CD8^+^ T cells. Data show n=4 individual mice/time point and treatment group from two independent experiments with mean ± SEM. Statistical significance: *P < 0.05, **P < 0.005, ***P < 0.001, one-way ANOVA with Tukey’s test for multiple comparisons.
